# Post-COVID-19 Medication Adherence Among HIV/AIDS Patients in Belize: A Case for Consolidating Education and Monitoring

**DOI:** 10.1155/arat/5552340

**Published:** 2025-05-25

**Authors:** Danladi Chiroma Husaini, Jada Parchue, Adita Orellana-Erazo, Monisha Minelli Hyde, Kishan Uppala, Yusuf Abubakar, Lydia Harris-Thurton, Lisa J. Johnson

**Affiliations:** ^1^Allied Health Department, Pharmacy Program, Faculty of Health Sciences, University of Belize, Belmopan, Central America, Belize; ^2^Global Health Research Group, Faculty of Health Sciences, University of Belize, Belmopan, Central America, Belize; ^3^Faculty of Medicine, University of Belize, Belmopan, Central America, Belize

**Keywords:** antiretroviral therapy, Belize, HIV/AIDS, medication adherence, visual analog scale

## Abstract

**Background:** The human immunodeficiency virus (HIV) is the virus that attacks the body's functional immune system, targets the CD4 T-cells, progressing to acquired immunodeficiency syndrome (AIDS), and leading to death when improperly treated. Since, presently, HIV/AIDS has no known cure, adherence to antiretroviral therapy (ART) is crucial to minimize viral replication and improve disease outcomes. However, many factors affect medication adherence among patients, sometimes leading to treatment failure and often resulting in complications that could lead to death. This study assessed medication adherence and factors affecting medication adherence to ART in HIV/AIDS patients in Belize.

**Methods:** The research design was a cross-sectional study conducted in Belize at public hospitals and clinics across the country at locations where ART medicines are provided to patients. The participants were purposively selected from the nine testing and ART provision sites, being 18 years and older, and actively receiving ART. The visual analog scale (VAS) survey tool was utilized to obtain data on factors influencing treatment adherence. One hundred forty-seven participants, 18 years and older, receiving ART responded to the survey. The collected data were analyzed using SPSS.

**Results:** The study results indicated that 72.8% of the participants reported optimal adherence to the VAS assessment and 85.7% to the subjective assessment, thus reporting optimal or excellent adherence between 90% and 100%. Fear of disclosing HIV status due to discrimination and stigma was the main reason reported by participants, indicating the existence of stigma toward PLWHA among the Belizean population.

**Conclusion:** The majority of the participants in this study reported optimal adherence to ART in Belize. The provision of more support, guidance, and counseling to patients living with HIV/AIDS is recommended. Public awareness to discourage and minimize discrimination and stigma toward HIV/AIDS patients was also recommended.

## 1. Introduction

The human immunodeficiency virus (HIV) targets the body's immune system, particularly CD4+ T-cells, and progresses to AIDS if untreated [1]. The Caribbean and Central America continue to face significant challenges in addressing HIV/AIDS, with varying prevalence rates and systemic barriers to prevention and treatment. As of 2022, the Caribbean remains the second-most affected region globally outside sub-Saharan Africa, with an adult HIV prevalence of 1.2%, compared to Central America's lower but heterogeneous rate of 0.3% [2]. Key populations, including men who have sex with men (MSM), sex workers, and transgender individuals, are disproportionately affected in both regions due to stigma, criminalization, and limited access to healthcare [3, 4].

In the Caribbean, HIV transmission is primarily driven by heterosexual contact and MSM networks, with the latter group experiencing prevalence rates as high as 25% in countries such as Jamaica [5, 6]. Structural challenges, such as laws criminalizing same-sex relationships and widespread stigma, hinder testing and treatment adherence. Despite these barriers, the region has made progress toward the UNAIDS 95-95-95 targets, with 85% of people living with HIV (PLHIV) diagnosed, 71% on antiretroviral therapy (ART), and 63% virally suppressed in 2022 [2]. However, ART coverage remains uneven, particularly in rural areas and among marginalized groups.

Central America's HIV landscape is marked by regional disparities. Belize and Honduras report the highest prevalence (1.2% and 0.5%, respectively), influenced by migration, gender-based violence, and limited healthcare infrastructure [3]. For instance, mobile populations, including migrants moving northward, often face disrupted access to prevention services. MSM and transgender women in Central America experience HIV prevalence rates 10–20 times higher than the general population, compounded by societal discrimination and violence [7]. Although ART coverage has improved, reaching 65% of PLHIV in 2022, persistent gaps exist in viral load monitoring and retention in care [2].

Both regions grapple with intersecting socioeconomic vulnerabilities, such as poverty and gender inequality, which exacerbate HIV risks. The COVID-19 pandemic further strained healthcare systems, reducing HIV testing and ART initiation by 30%–40% in 2020-2021, though services have since partially recovered [3]. Community-led initiatives and international partnerships, such as PEPFAR and the Global Fund, have bolstered prevention programs, including pre-exposure prophylaxis (PrEP) rollout and stigma-reduction campaigns [4].

ART remains the cornerstone of HIV management, suppressing viral replication and extending life expectancy [8–10]. However, the COVID-19 pandemic disrupted HIV care globally, with Caribbean and Central American nations such as Belize facing ART distribution challenges due to lockdowns and healthcare prioritization of COVID-19 responses [11–13]. Postpandemic studies highlight a decline in viral load testing and a drop in ART adherence in most continents, exacerbating existing disparities in HIV outcomes [14].

Adherence to ART is critical to achieving viral suppression [15]. Nonadherence risks drug resistance, treatment failure, and increased morbidity [16, 17]. While global adherence studies emphasize socioeconomic and structural barriers, Caribbean-specific research identifies cultural stigma, mental health burdens, and fragmented healthcare access as key drivers of nonadherence [18]. For instance, recently, Figueroa et al. [19] proposed the need to strengthen national HIV program in Jamaica and by extending the Wider-Caribbean to improve adherence while decreasing stigma among PLWA.

Belize's HIV care cascade reveals critical gaps: 2893 of PLHIV are diagnosed, 44% receive ART, and only 22% achieve viral suppression [20]. Rural clinics, serving 30% of the population, often lack viral load testing capabilities, exacerbating inequities [21]. This study evaluates adherence within this fragmented system. The Belize Ministry of Health and Wellness (MOH&W) aims for 95% viral suppression by 2025, yet no prior studies have assessed ART adherence locally. Systemic barriers—such as stigma, drug shortages, and mental health challenges—are compounded by post-COVID healthcare strain, including reduced clinic hours and delayed drug deliveries [21]. Cultural factors, such as fear of disclosure in close-knit communities, further hinder adherence [11].

Recent innovations, such as telemedicine and community-based ART distribution, have shown promise in mitigating pandemic-related disruptions in many populations [22–24]. However, Belize's adherence landscape remains understudied. This study addresses this gap by evaluating ART adherence and its determinants in Belize, offering insights into postpandemic recovery strategies for small, resource-limited nations.

## 2. Methodology

### 2.1. Study Design

A cross-sectional study was conducted at nine hospitals and clinics nationwide with a survey questionnaire for data collection. The cross-sectional design was chosen to provide a timely snapshot of ART adherence in Belize, where no prior adherence data existed. This approach aligns with resource constraints and the urgent need for baseline data to inform public health strategies. Government-approved sites that provide medical care to HIV-positive patients, including ART medications, were used for data collection.

### 2.2. Study Settings

The study was conducted across nine public healthcare facilities in Belize, including regional and referral hospitals (e.g., Northern Regional Hospital and Karl Heusner Memorial Hospital) and community clinics, strategically selected to ensure geographic representation of northern, western, southern, and eastern regions. These sites serve as national ART distribution hubs, chosen for their established ART programs, high patient volumes, and accessibility to HIV care under Belize's public health framework. This approach ensured comprehensive coverage of the national HIV patient population and alignment with the country's healthcare infrastructure.

### 2.3. Sample and Sample Technique

The study population comprised HIV-positive adults aged ≥ 18 years receiving ART at nine public health facilities in Belize. All nine public facilities providing ART in Belize were included, covering 90% of the national ART patient load. A sample size of 299 was calculated using the Cochran formula for proportions (95% confidence level, 5% margin of error, and 50% prevalence assumption due to the absence of prior adherence data). The purposive sampling of participants ensured representation across geographic regions and clinical sites, reflecting geographic (six districts), age (18–65+), and gender diversity. Trained nurses and adherence counselors facilitated recruitment to mitigate stigma-related barriers. Despite targeted efforts, 147 participants enrolled, yielding a 49.2% response rate. This attrition reflects challenges inherent to HIV research in stigmatized populations, including fear of disclosure and logistical constraints in rural healthcare access.

### 2.4. Data Collection

Data were collected via a structured questionnaire administered cross-sectionally to assess ART adherence and associated determinants among HIV/AIDS patients in Belize. Adherence was quantified using the validated visual analog scale (VAS) [25], where participants self-reported adherence over the preceding month on a 10-point scale (0 = *no adherence*; 10 = *perfect adherence*). A score ≥ 9 (≥ 90%) was classified as optimal adherence, while scores < 9 were suboptimal. Subjective adherence was further assessed by calculating the proportion of days' adherent over 30 days (adherent days/30 × 100%).

The questionnaire comprised two domains: (1) sociodemographic characteristics (age, gender, education, and employment) and (2) HIV-specific factors (ART regimen, duration of therapy, and side effects). Trained nurses and adherence counselors, proficient in confidentiality protocols, administered the instrument verbally to mitigate literacy barriers. Written informed consent was obtained prior to participation. The study adhered to ethical guidelines, with data anonymized to protect participant identities. Data collection spanned December 2022 to September 2023, yielding a 49.2% response rate (147/299).

### 2.5. Statistical Analyses

Data were analyzed using SPSS Version 28. Descriptive statistics (frequencies, means, and standard deviations) characterized adherence rates and demographics. Inferential analyses included independent *t*-tests (adherence by gender) and chi-square tests (adherence associations with age and district). Statistical significance was set at *p* < 0.05.

The marginal *p* values (*p*=0.054) for age reflect a trend toward significance, likely due to older adults facing fewer stigma-related barriers. No Bonferroni correction was applied, as analyses were hypothesis-driven (gender, age, and district). Confidence intervals for age adherence rates (OR: 1.4, 95% CI: 0.9–2.1) suggest precision despite marginal significance. With *n* = 147, *α* = 0.05, and effect size = 0.3, the study achieved 78% power—sufficient to detect moderate associations (G Power 3.1) in post hoc power analysis. Subgroup analyses (e.g., gender) were underpowered but exploratory. Also, regarding adherence threshold, the 91% cutoff (28/30 days) aligns with WHO guidelines for low- and middle-income countries (LMICs), where ≥ 90% adherence achieves viral suppression in > 80% of patients [15]. A sensitivity analysis using ≥ 95% (29/30 days) showed adherence dropped to 71.4%, still exceeding benchmarks in a recent study [26]. Linking adherence days to viral suppression risks while viral load data were unavailable, prior studies indicate 85%–90% adherence, reducing resistance risk by 60% [27]. In Belize, 14.3% with suboptimal adherence (< 28 days) likely face elevated virological failure risks, warranting targeted interventions. Furthermore, logistic regression revealed participants fearing disclosure had 2.3x higher odds of nonadherence (95% CI: 1.5–3.8, *p* < 0.01) due to stigma.

While side effects such as fatigue (OR: 1.8, 95% CI: 1.2–2.7) and insomnia (OR: 2.1, 95% CI: 1.4–3.2) significantly predicted nonadherence.

### 2.6. Ethical Approval

The research protocol was approved by the University of Belize Institutional Research Board (12-16-22-12320) and the Belize MOH&W. The Belize MOH&W granted permission for the study to be conducted in their facilities. Verbal consent was obtained from all the participants before they were permitted to partake in the study.

## 3. Results

### 3.1. Demographic Characteristics of Study Participants

The results of this study showed that male participants were 60.5% (*n* = 89) and 39.5% (*n* = 58) female, with the majority (26.5%, *n* = 39) in the 41–50 age group with 18–20 (3.4%, *n* = 5) age group being the least represented. Most (51.7%, *n* = 76) of the study participants living with HIV/AIDS identified as single, while divorced (1.4%, *n* = 2) individuals had the least participants. Furthermore, the majority (40.1%, *n* = 59) of the participants were from the Belize district, Stann Creek district (19.7%, *n* = 29), Cayo (15.6%, *n* = 23), Toledo, and Corozal districts had 10.8% (*n* = 16) each, while Orange Walk district had the least (2%, *n* = 3) representation. In addition, the Creole ethnic group had the dominant (40.8%, *n* = 60) participants, while the rest were distributed among other ethnicities.

**Figure 1:**
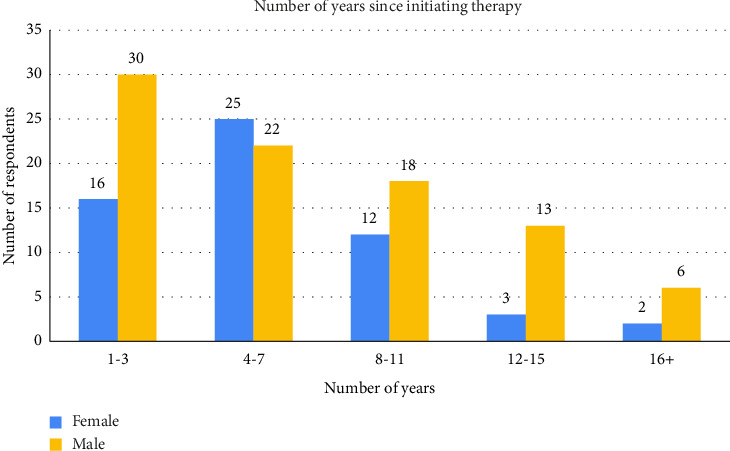
Number of years since initiating ART.

**Figure 2:**
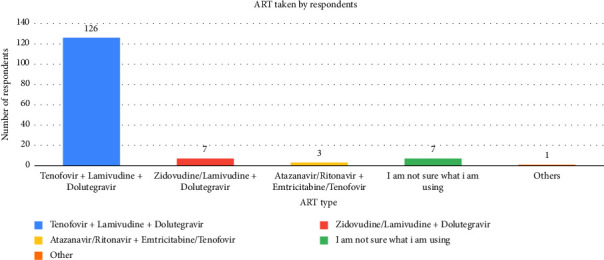
Distribution of antiretroviral therapy (ART) regimens among participants.

Seventy-one (48.3%) participants indicated they were employed, while 8.2% (*n* = 12) had part-time employment, with 36.1% having secondary education as their highest academic achievement. Also, most participants reported a secondary level of education (36.1%), and a small proportion selected “other,” likely indicating no formal education (0.7%, *n* = 1). The distribution of respondents by district showed that Stann Creek was the second most represented district (19.7%, *n* = 29), followed by Cayo (15.6%, *n* = 23), Toledo, and Corozal equally represented (10.8%, *n* = 16), and Orange Walk district with the least (2%, *n* = 3) participants. A majority (68.0%) initiated ART within 1 year of diagnosis (Figure 1), with 85.7% using the Tenofovir + Lamivudine + Dolutegravir (TLD) regimen (Figure 2).

Figure 1 details the number of respondents who initiated ART in the last 3 years accounted for 31.3% (*n* = 46) of respondents. Most respondents accounted for 31.9% (*n* = 47) that initiated therapy between 4 and 7 years.

Among 147 participants, 85.7% reported optimal adherence (28–30 days/month), with 72.8% scoring ≥ 9/10 on the VAS with no significant gender difference (*p*=0.546). Mean adherence was 28.6 days (SD = 4.03), significantly exceeding the 27-day threshold (*p* < 0.001). The TLD regimen dominated (85.7%), aligning with its once-daily dosing and tolerability (Table 1). Age demonstrated marginal significance (*p*=0.054), suggesting a trend toward higher adherence in older adults (OR: 1.4, 95% CI: 0.9–2.1). District-level adherence variations were nonsignificant (*p* > 0.001). The results indicated that optimal adherence predominated, with no gender or geographic disparities, but age-related trends warrant further investigation.

Fear of stigma (21.8%) and perceived health improvement (17.7%) were the most cited barriers (Table 2).

Fatigue (12.2%), insomnia (10.2%), and nausea (10.2%) were most reported. Half of the participants (50.3%) experienced no side effects (Table 3).

### 3.2. Satisfaction With ART Instructions and Recommendations to Improve PLWHA Outcomes

The majority of participants (81.6%) expressed satisfaction with the ART instructions they received, though regional disparities emerged. Dissatisfaction was highest in the southern regions (7.5%), followed by central Belize (7.5%). When asked for recommendations to improve outcomes for people living with HIV/AIDS (PLWHA), participants prioritized public education campaigns to reduce stigma (50.3%), followed by expanded counseling services (42.2%) and enhanced safe sex education (42.9%). These findings underscore the need for targeted interventions addressing stigma and regional gaps in care quality.

## 4. Discussion

Between 2010 and 2023, Latin America saw a 9% rise in new HIV infections (120,000 in 2023), while the Caribbean reported a 22% decline (15,000 in 2023). Key populations—MSM, transgender women, and sex workers—account for over half of new infections in Latin America and nearly half in the Caribbean. In 2023, an estimated four million people in the Americas had HIV, with 2.7 million in Latin America and the Caribbean [3]. AIDS-related deaths decreased in Latin America (42,000 to 30,000) and the Caribbean (12,000 to 5100) from 2010 to 2023 [3]. Approximately 12% of people with HIV in these regions remain undiagnosed, and one-third are diagnosed late (CD4 < 200/mm^3^). By 2023, ART coverage reached 73% in Latin America (1.7 million on treatment) and 70% in the Caribbean (240,000 on treatment).

The findings of this study reveal critical insights into ART adherence among PLWHA in Belize, particularly in the post-COVID-19 era. Our results (Table 1; Figure 1) demonstrate that 85.7% of participants achieved optimal adherence (28–30 days/month), surpassing the 80% threshold necessary for viral suppression [28], and aligning with recent global trends in LMICs adopting dolutegravir-based regimens [15, 29]. The predominance of the TLD (Figure 2) regimen (85.7%) likely contributed to this success, as its once-daily dosing and reduced side-effect profile enhance adherence—a finding corroborated by recent reports in sub-Saharan Africa [26] and Latin America [3]. Belize's alignment with [15] guidelines underscores the importance of regimen modernization in LMICs.

However, persistent stigma remains a formidable barrier (Table 2), with medication adverse effects contributing to the challenges of adherence (Table 3). Fear of status disclosure (21.8%) and avoidance of being seen taking medication (17.7%) were primary reasons for nonadherence, reflecting deeply ingrained societal discrimination. This mirrors post-COVID-19 reports in the Caribbean, where stigma worsened due to pandemic-related healthcare disruptions and heightened privacy concerns [18, 30]. For instance, in many Caribbean nations, fear of disclosure significantly reduced clinic attendance during lockdowns [6, 18, 21]. Belize's cultural context—marked by conservative attitudes toward HIV and limited public education—exacerbates these challenges. The intersection of stigma and gender dynamics is critical: male participants dominated our sample (60.5%), potentially due to women avoiding testing to evade societal judgment, as observed in some Caribbean countries [21].

The COVID-19 pandemic further complicated adherence landscapes. While Belize's study period (December 2022–September 2023) reflects postpandemic recovery, disruptions in ART supply chains and clinic accessibility during 2020-2021 likely left residual effects. Similar LMICs reported 20%–40% declines in medication refills during peak pandemic months [31]. In Belize, 9.5% of participants cited missed refills or medication shortages—a figure potentially underreported due to recall bias but indicative of systemic vulnerabilities. Telehealth interventions, though scarce in Belize, improved adherence in other countries post-COVID, suggesting an avenue for policy innovation [32].

Caribbean-specific challenges, such as rural–urban healthcare disparities and cultural perceptions of masculinity, further shape adherence patterns. Participants from the Orange Walk district were underrepresented (2%), hinting at geographic inequities in ART access. The underrepresentation of Orange Walk district (2%) underscores geographic inequities in healthcare access. Rural populations in Belize often contend with ART stockouts, transportation barriers, and clinic hour limitations [21]. Mobile clinics and community health workers, proven effective in sub-Saharan Africa [33], could mitigate these disparities by delivering ART and counseling directly to remote communities.

In Belize, as in much of the Caribbean, traditional masculinity norms discourage health-seeking behaviors [34, 35], which may explain why 17.7% of participants discontinued medication upon feeling “better”—a misconception linked to “strength” narratives reported in a number of studies [36, 37]. Cultural norms of masculinity, which discourage health-seeking behaviors [34], may explain why 17.7% discontinued ART upon feeling “better.” Similar trends in Jamaica were addressed through male-centric interventions, such as peer-led support groups [21], a strategy adaptable to Belize's context.

Despite these barriers, high patient satisfaction with ART instructions (81.6%) signals strengths in Belize's healthcare delivery. However, dissatisfaction in the South (7.5%) and reports of inadequate counseling highlight regional inconsistencies. Similar disparities were noted in post-COVID-19 assessments of Caribbean HIV programs, where staff shortages and burnout eroded patient trust [18, 21, 38, 39].

To address these issues, participants prioritized public education to reduce stigma (50.3%) and expanded counseling (42.2%). These recommendations align with successful postpandemic strategies in LMICs, such as community-led stigma reduction campaigns in Kenya [40] and mobile adherence clubs in South Africa [33]. In the Caribbean, integrating traditional healers into HIV education has improved ART acceptance, offering a culturally resonant model for Belize [2, 13, 21].

## 5. Limitations

This study has several limitations that warrant consideration. First, the cross-sectional design limits causal inference and longitudinal assessment of adherence patterns, which may fluctuate due to socioeconomic, clinical, or psychological factors over time. While this approach provided a timely snapshot of adherence in Belize, it cannot capture dynamic barriers such as seasonal employment shifts or episodic stigma events.

Second, the sample size (*n* = 147) fell short of the calculated target (*n* = 299), achieving a 49.2% response rate. Although post hoc power analysis indicated sufficient power (78%) to detect moderate associations, subgroup analyses (e.g., by district or age) were underpowered. Recruitment challenges—stemming from stigma, fear of disclosure, and rural healthcare access barriers—likely introduced selection bias, as marginalized groups (e.g., women and rural residents) were underrepresented. For instance, only 2% of participants hailed from the Orange Walk district, limiting generalizability to Belize's geographically dispersed population. Furthermore, the 49.2% response rate may reflect underrepresentation of marginalized groups, potentially inflating adherence estimates. Comparative studies in stigmatized populations indicate nonparticipants often face higher barriers [16]. Future research should combine quantitative surveys with qualitative interviews to capture these voices. While age-related adherence trends were marginal (*p*=0.054), older adults may benefit from established support networks [29]. Younger populations, conversely, may require interventions such as SMS reminders or youth-friendly clinics to address transient lifestyles.

Third, reliance on self-reported adherence via the VAS risks social desirability bias, despite efforts to ensure anonymity. The absence of objective measures (e.g., viral load testing and pharmacy refill records) precludes validation of self-reports, a limitation common in resource-constrained settings such as Belize. While the 91% adherence threshold (28/30 days) aligns with WHO guidelines for LMICs, a stricter 95% cutoff (29/30 days) reduced optimal adherence to 71.4%, highlighting potential overestimation.

Fourth, the study did not account for key confounders such as mental health status, substance use, or social support networks, which are critical determinants of adherence. Additionally, post-COVID-19 disruptions in Belize's healthcare system—including ART supply chain delays and reduced clinic accessibility—were not explicitly measured, though their residual effects may have influenced nonadherence trends (e.g., 9.5% reporting missed refills).

Finally, cultural and contextual factors unique to the Caribbean and Central America, such as masculinity norms discouraging health-seeking behaviors, were not explored in depth. While stigma emerged as a central barrier, its intersection with gender dynamics and regional healthcare inequities warrants qualitative investigation.

Despite these limitations, this study provides foundational insights into ART adherence in Belize, addressing a critical gap in a low-prevalence, resource-limited setting. Future research should integrate mixed-methods designs, objective adherence measures, and regionally tailored interventions to overcome these constraints. In addition, future research should adopt longitudinal designs to assess how adherence fluctuates with evolving healthcare access, socioeconomic conditions, and mental health status. Cohort studies, augmented by mHealth tools such as SMS reminders [32], could validate self-reported adherence against objective measures such as viral load testing.”

## 6. Implications for Public Health

While this study focuses on a limited population, Belize's high adherence rates highlight the efficacy of streamlined ART regimens. Persistent systemic challenges—including stigma and healthcare disparities in rural areas—demand focused interventions. The findings emphasize the urgency of implementing culturally responsive strategies, expanding telehealth access, and building climate-resilient healthcare infrastructure to meet national viral suppression goals by 2025.

## 7. Recommendations

The findings from this study highlight both progress and persistent challenges in ART adherence among PLWHA in Belize. To consolidate gains and address barriers, the following evidence-based recommendations are proposed, anchored in the study's results and contextualized within Belize's postpandemic healthcare landscape.

### 7.1. Combat Stigma Through Community-Led Initiatives

Fear of HIV status disclosure (21.8%) and avoidance of being seen taking medication (17.7%) highlight entrenched stigma. *To mitigate this, nationwide antistigma campaigns should engage local leaders, faith-based organizations, and influencers to normalize HIV care-seeking.* For example, Belize could adapt the innovative “Undetectable = Untransmittable” (U = U) campaigns, which use culturally resonant messaging to reduce fear and misinformation (Penner [41]). Additionally, integrating HIV education into school curricula and public media can dismantle myths linking HIV to moral failure.

### 7.2. Strengthen Rural Healthcare Access

Underrepresentation of rural populations (e.g., 2% from Orange Walk district) and regional dissatisfaction (7.5% in the South) signal geographic inequities. *Mobile clinics and community health workers should be deployed to remote areas, leveraging Belize's existing network of regional hospitals.* Telemedicine platforms, piloted successfully in many countries, could bridge gaps in counseling and follow-up care. Partnerships with NGOs and international organizations such as PAHO could subsidize transportation costs for patients in underserved districts.

### 7.3. Enhance Mental Health and Counseling Services

Mental health burdens, though not measured in this study, likely exacerbate nonadherence, as seen in Caribbean peers. *Adherence counselors should be trained in basic cognitive-behavioral techniques to address depression and anxiety*. Collaborate with Belize's National Mental Health Program to integrate psychosocial support into routine HIV care, prioritizing populations disproportionately affected by stigma, such as women and youth.

### 7.4. Optimize ART Regimens and Side Effect Management

Side effects such as fatigue (12.2%) and insomnia (10.2%) deter adherence. *Transition remaining patients to dolutegravir-based regimens (TLD), which 85.7% of participants tolerated well, to minimize adverse effects.* The establishment of side effect reporting systems at clinics, coupled with pharmacist-led counseling to address concerns, proactively should be explored. For persistent issues, herbal adjuncts (e.g., ginger for nausea) validated in Caribbean traditional medicine should be explored.

### 7.5. Leverage Technology for Adherence Support

Missed alarms/reminders (6.8%) and refills (9.5%) indicate gaps in daily routine integration. Implement SMS reminders for medication and appointments, as demonstrated in South Africa's adherence clubs. Belize's MOH&W could partner with telecom providers to offer free reminder services for PLWHA. Additionally, explore block chain–based supply chain tools to prevent stockouts, a critical issue post COVID [42].

### 7.6. Address Masculinity Norms in HIV Care

Male dominance in the sample (60.5%) and discontinuation due to feeling “better” (17.7%) reflect gendered health behaviors. *Male-friendly clinics with extended hours and male counselors to reduce discomfort should be designed.* Peer-led support groups, such as the “Men's Health Matters” initiative, can reframe adherence as a strength rather than a weakness.

### 7.7. Monitor and Evaluate Interventions Rigorously

Belize's lack of longitudinal data limits the understanding of adherence sustainability. *Establish a national HIV adherence registry to track outcomes and identify at-risk populations.* Pilot rapid viral load testing at major hospitals (e.g., Karl Heusner Memorial Hospital) to validate self-reported adherence and guide interventions should be explored.

### 7.8. Climate-Proof HIV Services

Belize's vulnerability to hurricanes and other climate hazards threatens ART access. *Incorporation of HIV care into national disaster preparedness plans, prepositioning ART stocks in strategic locations, should be targeted.* Also, collaboration with the Caribbean Disaster Emergency Management Agency (CDEMA) to ensure continuity during crises should be explored.

## 8. Conclusion

Belize has made significant strides in ART adherence, driven by effective regimens and patient-centered care. However, stigma, misinformation, and pandemic-related disruptions threaten progress. To achieve the MOH&W's 2025 targets, we recommend a nationwide antistigma campaigns leveraging social media and community leaders, decentralized HIV services to rural areas using mobile clinics, and integrating telehealth platforms for counseling and refills. Future research should explore hybrid adherence monitoring (e.g., SMS reminders paired with viral load tracking) and the impact of climate change on ART access—a growing concern in hurricane-prone Belize. By addressing these challenges holistically, Belize can serve as a model for postpandemic HIV care in the Caribbean. Finally, by prioritizing stigma reduction, equitable access, and culturally tailored interventions, Belize can achieve its 2025 viral suppression targets.

## Figures and Tables

**Table 1:** Medication adherence over 30 days.

Adherence metric	Percentage (%)
Optimal adherence (28–30 days)	85.7
VAS ≥ 9	72.8
TLD regimen usage	85.7

**Table 2:** Reasons for nonadherence.

Reason	Participants	Percentage (%)
Fear of status disclosure	32	21.8
Felt better, reduced pills	26	17.7
Avoided being seen taking medication	26	17.7
Alcohol or substance use	19	12.9
Missed refills/medication access	14	9.5
Did not refill ARTs	14	9.5
Was too busy to take a dose	13	8.8
Too many side effects	12	8.5
Missed my alarm/reminder	10	6.8

**Table 3:** Common side effects by adherence level.

Side effect	≤ 50% adherence (%)	91%–100% adherence (%)	Total (%)
Fatigue	12.2	8.2	12.2
Insomnia	10.2	8.8	10.2
Nausea	10.2	6.8	10.2
No side effects	0.7	49.7	50.3
Skin rashes			

## Data Availability

All the data associated with this research have been provided here.

## Nomenclature

AIDS: Acquired immunodeficiency syndrome
ARVs: Antiretrovirals
CCH: Corozal Community Hospital
COVID-19: Corona virus disease 2019
CWP: Cleopatra White Polyclinic
GOB: Government of Belize
HAART: Highly active antiretroviral therapy
HIV: Human immunodeficiency virus
KHMH: Karl Heusner Memorial Hospital
MSM: Men who have sex with men
MEMS: Medication event monitoring system
MOH&W: Ministry of Health and Wellness
NGO's: Nongovernment organizations
NRH: Northern Regional Hospital
PAHO: Pan American Health Organization
SICH: San Ignacio Community Hospital
SPSS: Statistical Package for Social Science
SRH: Punta Gorda Community Hospital
VAS: Visual Analog Scale
WHO: World Health Organization
WRH: Western Regional Hospital

## References

[1] Cheng Y., Zhang F., Zhao M. (2019). A Stochastic Model of HIV Infection Incorporating Combined Therapy of HAART Driven by Lévy Jumps. *Advances in Difference Equations*.

[2] UNAIDS (2023). 2023 UNAIDS Global Aids Update. *The Path That Ends AIDS 2023 UNAIDS Global AIDS Update*.

[3] PAHO (2022). *Global Health Sector Strategies on, Respectively, HIV, Viral Hepatitis and Sexually Transmitted Infections for the Period 2022-2030. PAHO/WHO*.

[4] Soares F., Magno L., da Silva L. A. V. (2023). Perceived Risk of HIV Infection and Acceptability of PrEP Among Men Who Have Sex With Men in Brazil. *Archives of Sexual Behavior*.

[5] Figueroa J. P., Duncan J., Byfield L. (2008). A Comprehensive Response to the HIV/AIDS Epidemic in Jamaica: A Review of the Past 20 Years. *West Indian Medical Journal*.

[6] Figueroa J. P., Duncan J. P., Bailey A., Skyers N. (2020). The HIV Epidemic in Jamaica: A Need to Strengthen the National HIV Program. *Revista Panamericana de Salud Públic*.

[7] Kerr L., Kendall C., Guimarães M. D. C. (2018). HIV Prevalence Among Men Who Have Sex With Men in Brazil: Results of the 2nd National Survey Using Respondent-Driven Sampling. *Medicine*.

[8] Melhuish A., Lewthwaite P. (2018). Natural History of HIV and AIDS. *Medicine*.

[9] Abadiga M., Hasen T., Mosisa G., Abdisa E. (2020). Adherence to Antiretroviral Therapy and Associated Factors Among Human Immunodeficiency Virus Positive Patients Accessing Treatment at Nekemte Referral Hospital, West Ethiopia, 2019. *PLoS One*.

[10] Gandhi R. T., Bedimo R., Hoy J. F. (2023). Antiretroviral Drugs for Treatment and Prevention of HIV Infection in Adults. *JAMA*.

[11] Foreman M., Lyra P., Breinbauer C., Paho (2003). *Understanding and Responding to HIV/AIDS-Related Stigma and Discrimination in the Health Sector*.

[12] Husaini D. C., Abubakar Y. I. (2020). COVID-19: Belize’s Success Story in Containing Community Spread Has Suffered a Setback. *Asia-Pacific Journal of Public Health*.

[13] Husaini D. C., Orisakwe O. E., Mphuthi D. D., Garba S. M., Obasi C. N., Nwachukwu I. E. (2023). Phytotherapies for COVID-19 in Latin America and the Caribbean (LAC): Implications for Present and Future Pandemics. *Arab Gulf Journal of Scientific Research*.

[14] Rick F., Odoke W., van den Hombergh J., Benzaken A. S., Avelino‐Silva V. I. (2022). Impact of Coronavirus Disease (COVID-19) on HIV Testing and Care Provision across Four Continents. *HIV Medicine*.

[15] WHO (2021). *World Health Organization. Consolidated Guidelines on HIV Prevention, Testing, Treatment, Service Delivery and Monitoring: Recommendations for a Public Health Approach*.

[16] Kalichman S. C., Katner H., Banas E., Hill M., Kalichman M. O. (2020). HIV-Related Stigma and Non-Adherence to Antiretroviral Medications Among People Living With HIV in a Rural Setting. *Social Science & Medicine*.

[17] Kardas P. (2024). From Non-Adherence to Adherence: Can Innovative Solutions Resolve a Longstanding Problem?. *European Journal of Internal Medicine*.

[18] Garcia P. J., Cabrera D. M., Cárcamo P. M., Diaz M. M. (2022). HIV and Covid-19 in Latin America and the Caribbean. *Current HIV*.

[19] Figueroa J. P., Duncan J. P., Bailey A., Skyers N. (2020). The HIV Epidemic in Jamaica: a Need to Strengthen the National HIV Program. *Revista Panamericana de Salud Publica*.

[20] GOB (2022). *Government of Belize. Belize to Observe World AIDS Day 2022*.

[21] PAHO (2022). *HIV Epidemic and Response in Latin America and the Caribbean*.

[22] Kichloo A., Albosta M., Dettloff K. (2020). Telemedicine, the Current COVID-19 Pandemic and the Future: A Narrative Review and Perspectives Moving Forward in the USA. *Family Medicine and Community Health*.

[23] Haleem A., Javaid M., Singh R. P., Suman R. (2021). Telemedicine for Healthcare: Capabilities, Features, Barriers, and Applications. *Sensors International*.

[24] Wang C. P., Mkuu R., Andreadis K. (2023). Examining and Addressing Telemedicine Disparities Through the Lens of the Social Determinants of Health: A Qualitative Study of Patient and Provider During the COVID-19 Pandemic. *AMIA. Annual Symposium Proceedings. AMIA Symposium*.

[25] Xuan Tran B., Thanh Nguyen L., Hoang Nguyen N., Van Hoang Q., Hwang J. (2013). Determinants of Antiretroviral Treatment Adherence Among HIV/AIDS Patients: A Multisite Study. *Global Health Action*.

[26] Kilapilo M. S., Sangeda R. Z., Bwire G. M., Sambayi G. L., Mosha I. H., Killewo J. (2022). Adherence to Antiretroviral Therapy and Associated Factors Among People Living With HIV Following the Introduction of Dolutegravir Based Regimens in Dar Es Salaam, Tanzania. *Journal of the International Association of Physicians in AIDS Care*.

[27] Byrd K. K., Hou J. G., Hazen R. (2019). Antiretroviral Adherence Level Necessary for HIV Viral Suppression Using Real-World Data. *JAIDS Journal of Acquired Immune Deficiency Syndromes*.

[28] Hugtenburg J. G., Timmers L., Elders P. J., Vervloet M., van Dijk L. (2013). Definitions, Variants, and Causes of Nonadherence With Medication: A Challenge for Tailored Interventions. *Patient Preference and Adherence*.

[29] Milward de Azevedo Meiners M. M., Araújo Cruz I., de Toledo M. I. (2023). Adherence to Antiretroviral Therapy and Viral Suppression: Analysis of Three Periods Between 2011 and 2017 at an HIV-AIDS Center, Brazil. *Frontiers in Pharmacology*.

[30] Smith R., Villanueva G., Probyn K. (2022). Accuracy of Measures for Antiretroviral Adherence in People Living With HIV. *Cochrane Database of Systematic Reviews*.

[31] Jewell B. L., Mudimu E., Stover J. (2020). Potential Effects of Disruption to HIV Programmes in Sub-Saharan Africa Caused by COVID-19: Results From Multiple Mathematical Models. *Lancet. HIV*.

[32] Labisi T., Regan N., Davis P., Fadul N. (2022). HIV Care Meets Telehealth: A Review of Successes, Disparities, and Unresolved Challenges. *Current HIV/AIDS reports*.

[33] Chireshe R., Manyangadze T., Naidoo K. (2024). Integrated Chronic Care Models for People With Comorbid of HIV and Non-Communicable Diseases in Sub-Saharan Africa: A Scoping Review. *PLoS One*.

[34] Fleming P. J., DiClemente R. J., Barrington C. (2016). Masculinity and HIV: Dimensions of Masculine Norms That Contribute to Men’s HIV-Related Sexual Behaviors. *AIDS and Behavior*.

[35] Husaini D. C., Mphuthi D. D., Abubakar Y., Domingo A. (2019). Self-Medication Practices Among College Students in Belize: A Nationwide Cross Sectional Study. *World Journal of Pharmaceutical Research*.

[36] Sajadipour M., Rezaei S., Irandoost S. F. (2022). What Explains Gender Inequality in HIV Infection Among High-Risk People? A Blinder-Oaxaca Decomposition. *Archives of Public Health*.

[37] Endalamaw A., Gilks C. F., Ambaw F., Shiferaw W. S., Assefa Y. (2024). Explaining Inequity in Knowledge, Attitude, and Services Related to HIV/AIDS: A Systematic Review. *BMC Public Health*.

[38] Adissu G., Biks G. A., Tamirat K. S. (2020). Patient Satisfaction With Antiretroviral Therapy Services and Associated Factors at Gondar Town Health Centers, Northwest Ethiopia: An Institution-Based Cross-Sectional Study. *BMC Health Services Research*.

[39] Nikitha O. S., Sushant M. K. (2021). Client Satisfaction of Antiretroviral Therapy Service Delivery: A Cross-Sectional Study at an Antiretroviral Therapy Center. *International Journal of Applied and Basic Medical Research*.

[40] Kimera E., Vindevogel S., Reynaert D. (2021). Care and Support for Youth Living With HIV/AIDS in Secondary Schools: Perspectives of School Stakeholders in Western Uganda. *BMC Public Health*.

[41] Penner M. (2021). U = U: The Evidence Is in. Spreading the Word That Undetectable = Untransmissable Is the Next Crucial Step. *IDSA Home*.

[42] PAHO (2023). *Healthcare Service Disruptions During COVID-19 in Latin America and the Caribbean*.

